# Utilization of Airway Pressure Release Ventilation as a Rescue Strategy in COVID-19 Patients: A Retrospective Analysis

**DOI:** 10.1177/08850666211030899

**Published:** 2021-07-07

**Authors:** Omar Mahmoud, Deep Patadia, James Salonia

**Affiliations:** 1 Department of Medicine, Icahn School of Medicine at Mount Sinai, Mount Sinai Morningside and Mount Sinai West Hospital, New York, NY, USA; 2 Division of Pulmonary and Critical Care, Icahn School of Medicine at Mount Sinai, Mount Sinai Morningside, Mount Sinai West, and Mount Sinai Beth Israel Hospital, New York, NY, USA

**Keywords:** mechanical ventilation, respiratory failure, intensive care unit

## Abstract

**Background:**

Airway Pressure Release Ventilation (APRV) is a pressure controlled intermittent mandatory mode of ventilation characterized by prolonged inspiratory time and high mean airway pressure. Several studies have demonstrated that APRV can improve oxygenation and lung recruitment in patients with Acute Respiratory Distress Syndrome (ARDS). Although most patients with COVID-19 meet the Berlin criteria for ARDS, hypoxic respiratory failure due to COVID-19 may differ from traditional ARDS as patients often present with severe, refractory hypoxemia and significant variation in respiratory system compliance. To date, no studies investigating APRV in this patient population have been published. The aim of this study was to evaluate the effectiveness of APRV as a rescue mode of ventilation in critically ill patients diagnosed with COVID-19 and refractory hypoxemia.

**Methods:**

We conducted a retrospective analysis of patients admitted with COVID-19 requiring invasive mechanical ventilation who were treated with a trial of APRV for refractory hypoxemia. PaO_2_/FIO_2_ (P/F ratio), ventilatory ratio and ventilation outputs before and during APRV were compared.

**Results:**

APRV significantly improved the P/F ratio and decreased FIO_2_ requirements. PaCO_2_ and ventilatory ratio were also improved. There was an increase in tidal volume per predicted body weight during APRV and a decrease in total minute ventilation. On multivariate analysis, higher inspiratory to expiratory ratio (I: E) and airway pressure were associated with greater improvement in P/F ratio.

**Conclusions:**

APRV may improve oxygenation, alveolar ventilation and CO_2_ clearance in patients with COVID-19 and refractory hypoxemia. These effects are more pronounced with higher airway pressure and inspiratory time.

## Introduction

The novel Coronavirus Disease 2019 (COVID-19) caused by the severe acute respiratory syndrome coronavirus 2 (SARS-CoV-2) has become a global health emergency and has created unprecedented challenges to health care systems worldwide. As of March 2021, this disease is responsible for 120 million infections and has led to 2.7 million deaths worldwide. When admitted to the intensive care unit (ICU), patients often require a high level of care complicated by severe hypoxemia and high risk of death. A recent meta-analysis estimated a mortality rate of 41.6% among patients with COVID-19 admitted to the intensive care unit (ICU).^1^

Airway Pressure Release Ventilation (APRV) is mode of ventilation characterized by the application of continuous positive airway pressure (P_high_) maintained for a preset inspiratory time (T_high_) and intermittent decompressions to a lower pressure (P_low_) for a shorter expiratory time (T_low_). The inverse ratio ventilation facilitates lung recruitment, increases the respiratory system compliance, and improves gas exchange and oxygenation when compared to a traditional, non-inverse-ratio ventilatory strategy.^2^ However, the lack of standardized protocols and the scarcity of clinical evidence from prospective studies have made APRV an infrequently used mode of ventilation that is mostly reserved as a rescue ventilatory strategy in patients with refractory hypoxemia and acute respiratory distress syndrome (ARDS).

Although most patients with COVID-19 meet the Berlin criteria for ARDS, this clinical syndrome is substantially different from the traditional ARDS as patients often present with severe, refractory hypoxemia and significant variation in respiratory system compliance.^3^ Therefore, it remains unclear if the rescue strategies implemented in ARDS have a role in acute hypoxemic respiratory failure due to COVID-19.

The aim of this study was to assess the effectiveness of APRV as a rescue mode of ventilation for refractory hypoxemia in patients with COVID-19.

## Materials and Methods

The Mount Sinai Health System is an integrated network of 8 hospitals, which serves a large and diverse population in the New York metropolitan area. The study is a retrospective analysis of patients with COVID-19 and respiratory failure admitted to the intensive care units at the Mount Sinai Health System from January 1 to June 30, 2020. We reviewed our hospital medical record system to identify patients who were at least 18 years of age, had a positive SARS-CoV-2 PCR from nasopharyngeal swab and were intubated and invasively ventilated for acute respiratory failure.

We included patients who developed refractory hypoxemia, defined as arterial partial pressure of oxygen (PaO_2_) to fractional inspired oxygen (FIO_2_) ratio (P/F ratio) less than 200 when supported with a positive end-expiratory pressure (PEEP) of 5 cm H_2_O or greater and a FIO_2_ of at least 70%, who were transitioned to APRV as an alternative ventilatory strategy for a minimum of 8 hours. APRV settings were variable and at the discretion of the practicing physician. Ventilatory settings and outputs and arterial blood gas analysis (ABG) within 6 hours before and during APRV were recorded. We excluded patients with evidence of cardiogenic pulmonary edema, those treated with extracorporeal membrane oxygenation (ECMO), and those on mechanical ventilation for less than 24 hours.

We collected data regarding patients’ demographics, vital signs, use of antibiotics, corticosteroids, anticoagulants, pulmonary vasodilators, neuro-muscular blocking agents (NMBAs) and prone positioning. The P/F ratios before and during APRV were compared to determine the effects of APRV on oxygenation.

Since the end tidal carbon dioxide (ETCO_2_) was recorded in only 17 patients, we utilized the ventilatory ratio as a surrogate of dead space fraction, as described by other authors.^4^ We compared the ventilatory ratio before and during APRV to study the effects of APRV on alveolar dead space and carbon dioxide (CO_2_) clearance.

Inspiratory and expiratory times for APRV were not available in our database and only the inspiratory to expiratory time ratio (I: E ratio) was utilized in our analysis.

Plateau pressures are not routinely recorded in our medical record system and therefore the determination of static compliance before and during APRV trial was not possible. Instead, dynamic compliance was utilized to evaluate the effects of APRV on respiratory system compliance.

The Shapiro–Wilk test was used to test for normal distribution. Parametric data were reported as mean ± standard deviation whereas nonparametric data were presented as median and interquartile range (IQR). Student’s t-test and Wilcoxon signed-rank test were used to compare parametric and nonparametric data, respectively. Multivariate analysis was utilized to study the correlation between inspiratory to expiratory time ratio (I: E), airway pressure and change in P/F ratio. All statistical analysis was performed using software STATA. A *P*-value of 0.05 or less was considered valid for statistical significance.

The study protocol was approved by the Institutional Review Board and the COVID-19 Review Committee of the Icahn School of Medicine at Mount Sinai and a waiver of informed consent was granted.

## Results

### Patient Characteristics

The baseline characteristics of the 60 patients enrolled in this study are summarized in Table 1.

**Table 1.** Baseline Characteristics of the Patients.

Patient characteristics n (%)	
No.	60
Age (Years)	65 ± 12
Female	22 (36.66)
Male	38 (63.33)
BMI (kg/m^2^)	30.84 (25.62 to 34.99)
Race	
African American	18 (30)
Asian	5 (8.33)
Caucasian	6 (10)
Haitian	5 (8.33)
Jamaican	2 (3.33)
Other	24 (40)
Comorbidities	
Median number of comorbidities	2.21 (1.78 to 2.65)
Patients without comorbidities	8 (13.33)
Hypertension	34 (56.66)
Hyperlipidemia	14 (23.33)
Diabetes mellitus	28 (46.66)
Lung disease	13 (21.66)
Cardiovascular disease	17 (28.33)
Renal disease	6 (10.00)
Malignancy	6 (10.00)
Length of hospital stay (Days)	19.5 (11.5 to 36.5)
Mortality	48 (80)
Continuous sedation	57 (95)
Antibiotics	45 (75)
Hydroxychloroquine	17 (28.33)
Corticosteroids	32 (53.33)
Anticoagulation	57 (95)
Systolic blood pressure (mm Hg)	122 ± 25
Diastolic blood pressure (mm Hg)	64 ± 13
Heart rate (bpm)	95 ± 19
Temperature (°F)	99.20 ± 1.34
White blood cell count (10^3^/µL)	14.55 (10.55 to 21.2)
C-reactive protein (mg/L)	160 (58 to 264)
D-dimer (mcg/mL FEU)	5.57 (2.77 to 14.8)
RASS score	−3 (−4 to −2)

Abbreviations: FEU, fibrinogen equivalent units; RASS, Richmond agitation sedation scale.

Forty-eight patients (80%) died in the hospital. The mean age was 65 ± 12 years, 22 patients (37%) were female. Patients presented with several comorbid conditions and only 8 (13.3%) had no comorbidities. Most of the patients were classified as overweight or obese (median BMI: 30.84, interquartile range 25.62-34.99) and were hospitalized for a median of 19 days. The majority of the patients were African American (30%) followed by Caucasian (10%) and Asian (8.33%).

Almost all the patients (95%) received systemic anticoagulation and 32 (53%) were treated with corticosteroids within 48 hours of APRV trial. The mean systolic and diastolic arterial pressures at the time of APRV trial were 122 mm Hg and 64 mm Hg, respectively.

The median serum C-reactive protein (CRP) and D-dimer levels immediately prior to APRV trial were 160 mg/dL and 5.57 mcg/mL FEU (fibrinogen equivalent units), respectively.

### Mechanical Ventilation Outputs

Mechanical ventilation outputs are reported in Table 2. Patients remained intubated and supported with mechanical ventilation for a median of 14 days. The median number of days from admission to intubation was 6 and patients were transitioned to APRV after a median of 5 days of conventional mechanical ventilation.

**Table 2.** Mechanical Ventilation Outputs.

Mechanical ventilation outputs n (%)		
	Duration of mechanical ventilation (days)	14 (8-24)
	Time from admission to intubation (days)	6 (2-11)
	Time from intubation to APRV trial (days)	5 (2-11)
Before APRV trial	Mechanical ventilation mode	
	Volume control (VC)	42 (70)
	Pressure control (PC)	5 (8.33)
	Pressure regulated volume control (PRVC)	11 (18.33)
	PEEP (cm H_2_O)	11.38 ± 3.71
	FIO_2_ (%)	100 (75-100)
	PIP (cm H_2_O)	34.5 (27-40)
	I: E	0.69 ± 0.33
	TV (mL)	421 ± 89.56
	TV/PBW (mL/Kg)	6.76 ± 1.70
	Minute ventilation (L/min)	12.39 ± 2.99
	Prior trial of other rescue therapies	
	Prone positioning	40 (68.97)
	Pulmonary vasodilators	9 (15)
	Paralytics	25 (41.65)
During APRV trial	PEEP (cm H_2_O)	5 (3.8-8)
	FiO_2_ (%)	80 (60-100)
	PIP (cm H_2_O)	34.09 ± 7.27
	I: E	4 (3-5.7)
	TV (mL)	525.78 ± 188.68
	TV/PBW (mL/Kg)	7.86 (7.06-9.85)
	Minute ventilation (L/min)	10.87 ± 3.11
	Continuation of other rescue therapies	
	Prone positioning	22 (37.93)
	Pulmonary vasodilators	2 (3.33)
	Paralytics	22 (37.93)
	Duration of APRV (Hours)	40 (24-96)

Abbreviations: PEEP, positive end expiratory pressure; FIO_2_, fraction of inspired oxygen; PIP, peak inspiratory pressure; TV, tidal volume; I: E, inspiratory to expiratory ratio; PBW, predicted body weight.

Before APRV trial, the majority of the patients were ventilated using volume control mode (70%) with a mean PEEP of 11 cm H_2_O and median FIO_2_ of 100%. The mean tidal volume per predicted body weight (TV/PBW) was 6.76 mL/Kg and the median peak inspiratory pressure (PIP) was 34.5 cm H_2_O. The mean I: E ratio and minute ventilation were 0.69 and 12.39 L/min, respectively.

During conventional mechanical ventilation, most of the patients were also treated with other rescue strategies for hypoxemia. Forty patients (69%) were placed in prone position, 9 (15%) received treatment with pulmonary vasodilators (3 patients received nitric oxide and 6 received inhaled epoprostenol) and 25 patients (41.65%) were treated with NMBAs.

These rescue strategies were started at any point during conventional mechanical ventilation and were either discontinued before APRV trial or continued during APRV.

During APRV, 22 patients (37.93%) continued to be in prone position, 2 patients continued to receive pulmonary vasodilators (1 patient epoprostenol and 1 nitric oxide) and 22 patients (37.93%) were paralyzed. None of the patients received any additional rescue treatment that was not previously started during conventional mechanical ventilation.

The median duration of APRV trial was 40 hours and the median I: E ratio was 4. During APRV, the mean PIP and TV/PBW were 34 cm H_2_O and 7.86 mL/Kg, respectively.

### Study Outcomes

The primary outcomes of the study are presented in Table 3. The P/F ratio significantly improved during APRV trial (103 vs 131.75), oxygen requirements decreased (median FIO_2_ before and during APRV 100 and 80, respectively) and the PaO_2_ improved (80 mm Hg vs 91.5 mm Hg before and during APRV, respectively). There was no change in arterial pH with APRV.

**Table 3.** Study Outcomes.

	Before APRV	During APRV	*P* value
PaO_2_/FIO_2_ ratio	103 (75-154.23)	131.75 (94.15-221)	0.0001
FiO_2_ (%)	100 (75-100)	80 (60-100)	0.0034
pH	7.265 (7.16-7.39)	7.31 (7.25-7.38)	0.0736
PaO_2_ (mm Hg)	80 (65-103)	91.5 (76-135.5)	0.0072
PaCO_2_ (mm Hg)	54 (42-73)	45.8 (41-56.75)	0.0051
TV (mL)	421.93 ± 89.56	525.78 (188.68)	<0.0001
TV/PBW (mL/Kg)	6.58 (5.69-7.86)	7.86 (7.06-9.85)	<0.0001
Minute ventilation (L/min)	12.39 ± 2.99	10.87 ± 3.11	0.0005
Ventilatory ratio	2.85 (2.07-3.85)	2.24 (1.72-2.72)	0.0054
Dynamic compliance (mL/cm H_2_O)	21.07 (13.33-25.42)	19.25 (14.14-24.65)	0.3324

Abbreviations: TV, tidal volume; PBW, predicted body weight.

We also found that during APRV patients had a reduction in PaCO_2_ (54 mm Hg vs 45.8 mm Hg), minute ventilation (12.39 L/min vs 10.87 L/min) and ventilatory ratio (2.85 vs 2.24). TV/PBW was increased during APRV (7.86 mL/Kg vs 6.58 mL/Kg). Dynamic compliance did not significantly differ before and during the APRV trial.

**Figure 1.**
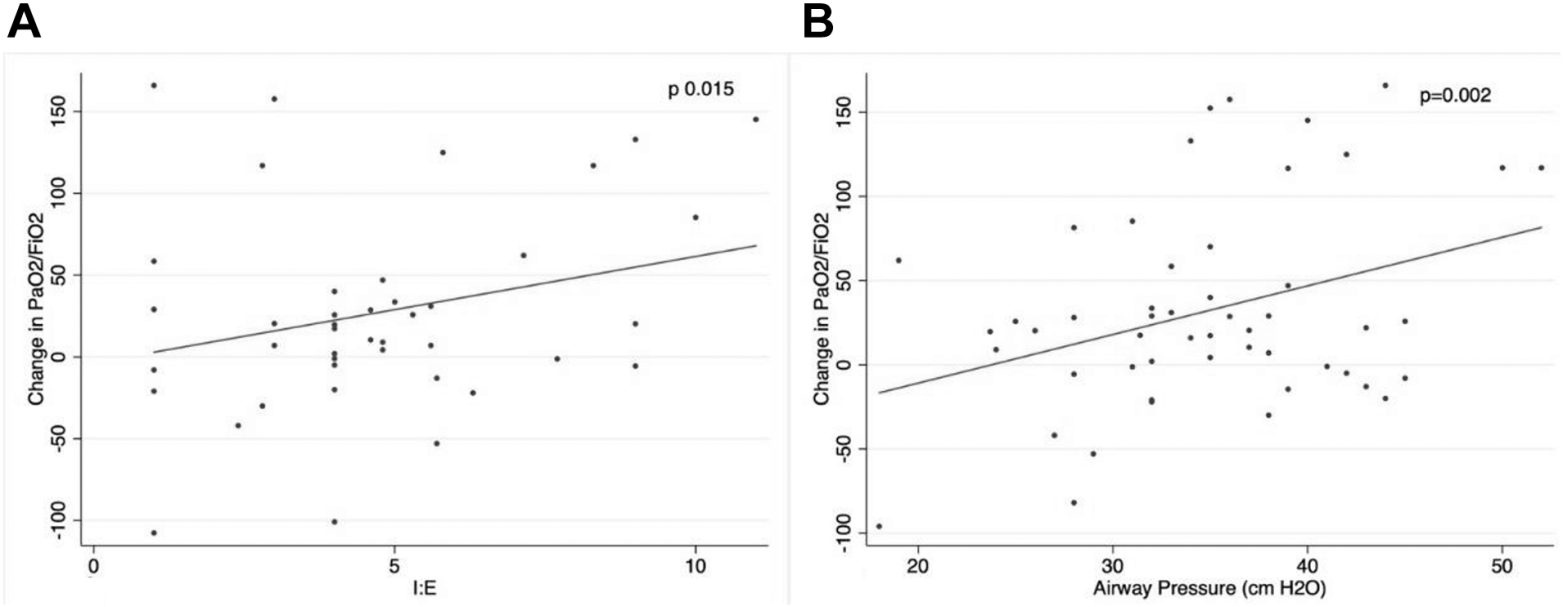
Graphic representation of multivariate analysis: (A) Correlation between I: E ratio and change in P/F ratio before and during APRV. For every 1 unit increase in the I: E ratio, the P/F ratio increases by 10.127, *P* = 0.015; (B) Correlation between airway pressure and change in P/F ratio before and during APRV. For every 1 cm H_2_O increase in airway pressure, the P/F ratio increases by 4.314, *P* = 0.002.

We performed multivariate analysis to assess the effects of I: E ratio and airway pressure on oxygenation. Results are shown Figure 1. After adjustment for confounders, airway pressure was found to be linearly correlated to the change in P/F ratio before and during APRV (for every 1 cm H_2_O incremental increase in airway pressure, the P/F ratio increases by 4.314, *P* = 0.02). The I: E ratio was also correlated to a greater change in P/F ratio (P/F ratio increases by 10.127 for every 1 unit increase in the I: E ratio, *P* = 0.015).

### Survival Analysis

Table 4 summarizes the clinical characteristics of survivors and deceased patients. Compared to survivors, patients who died in the hospital were significantly older (mean age 68 years and 54 years in non-survivors and survivors, respectively). Non-survivors presented with significantly lower P/F ratio (92 and 142.4 in non-survivors and survivors, respectively) and higher ventilatory ratio (3.24 and 2.49 in non-survivors and survivors, respectively). There was no difference in dynamic compliance, PEEP, PIP, use of NMBAs, antibiotics, corticosteroids, vasopressors and prior trial of prone positioning.

**Table 4.** Survival Analysis.

Survival analysis, n (%)				
		Survivors (n = 12)	Non survivors (n = 48)	*P* value
	Age (years)	54 ± 13	68 ± 10	<0.0001
	Female	3 (25%)	19 (39.58%)	0.3571
	BMI (kg/m^2^)	30.83 (22.4-31.75)	31.05 (26.88-36)	0.2661
	Number of comorbidities	2 (1-2.5)	2 (1-3)	0.3849
	Duration of mechanical ventilation (Days)	17 (8-24)	14 (7-24)	0.8194
	Time from admission to intubation (Days)	2 (1-6)	6 (2-11)	0.0603
	Time from intubation to APRV trial (days)	6 (2-13)	5 (2-9)	0.3155
	Prone positioning	7 (58.3)	33 (68.75%)	0.6778
	NMBAs	3 (25%)	22 (48.83%)	0.1967
	Vasopressors	7 (58.3%)	34 (70.83%)	0.4137
	Corticosteroids	7 (58.3)	25 (52.1%)	0.7003
	Antibiotics	3 (25%)	22 (45.83%)	0.1942
	Hydroxychloroquine	5 (41.66%)	12 (25%)	0.2558
	WBC (10^3^/µL)	8.6 (6.61-12.05)	16.75 (12.35-21.9)	0.0010
	C reactive protein (mg/L)	169.86 (31-258.1)	160.10 (60.71-264)	0.8295
	D-dimer (mcg/mL FEU)	3.32 (2.78-7.50)	5.97 (2.75-15.12)	0.2954
Before APRV trial	PaO_2_/FIO_2_ ratio	142.4 (103-170.75)	92 (69-124)	0.0341
	PEEP (cm H_2_O)	10.69 ± 3.2856	11.55 ± 3.825	0.4776
	PIP (cm H_2_O)	31.1 ± 8.33	34.92 ± 9.33	0.2032
	TV/PBW (mL/Kg)	6.91 ± 1.14	6.73 ± 1.82	0.7570
	Dynamic compliance (mL/cm H_2_O)	24.60 (17.58-27.82)	20.87 (12.73-24.87)	0.1555
	Ventilatory ratio	2.49 ± 0.90	3.24 ± 1.29	0.0383
During APRV trial	PaO_2_/FIO_2_ ratio	166.25 (122-284.75)	119.71 (86.5-212)	0.0760
	PEEP (cm H_2_O)	6.15 (3.9-8.3)	5 (3.8-7.7)	0.5474
	PIP (cm H_2_O)	29.275 ± 7.03	35.32 ± 6.88	0.0045
	TV/PBW (mL/Kg)	7.17 (6.85-7.85)	7.99 (7.12-9.99)	0.1850
	Dynamic compliance (mL/cm H_2_O)	25.60 (16.93-35.60)	18.97 (13.72-22.46)	0.0555
	Ventilatory ratio	2 (1.65-2.47)	2.40 (1.79-2.87)	0.2395

Abbreviations: BMI, body mass index; NBMAs, neuro-muscular blocking agents; WBC, white blood cells; FEU, fibrinogen equivalent units; PEEP, positive end expiratory pressure; PIP, peak inspiratory pressure; TV/PBW, tidal volume per predicted body weight.

During APRV ventilation, survivors had lower inspiratory pressures (29 cm H_2_O and 35 cm H_2_O in survivors and non-survivors, respectively). We did not observe a statistically significant difference in P/F ratio (166.25 vs 119.71, *P =* 0.0760), dynamic compliance (25.60 mL/cm H_2_O vs 18.97 mL/cm H_2_O, *P =* 0.0555) or TV/PBW (7.17 mL/Kg vs 7.99 mL/Kg, *P* = 0.1850) between survivors and non-survivors during APRV. D-dimer and CRP did not differ between the 2 groups. However, patients who died in the ICU had a significantly higher white blood cell count (8.6 103/µL vs 16.75 103/µL in survivors and non-survivors respectively, *P =* 0.0010).

There was no difference in duration of mechanical ventilation and time of implementation of APRV in survivors and non-survivors.

## Discussion

The management of ARDS and refractory hypoxemia due to COVID-19 infection poses significant clinical challenges. When conventional methods of mechanical ventilation fail to achieve adequate oxygenation and ventilation goals, alternative modes of ventilation need to be utilized. The aim of this study was to assess the physiological changes in patients with severe ARDS secondary to COVID-19 undergoing a trial of APRV for refractory hypoxemia.

The etiology of hypoxemia in COVID-19 has not been fully elucidated. Several mechanisms have been proposed including the development of intrapulmonary shunting, intravascular thrombosis with increased dead space ventilation, and ventilation-perfusion mismatch due to hypoxic pulmonary vasoconstriction.^5^ The formation of microvascular thrombosis seems to play an important role in the pathogenesis of the disease as demonstrated in autopsy studies.^6^

In this retrospective cohort study, we showed that APRV led to a substantial improvement in P/F ratio and PaO_2_ and decreased FiO_2_ requirements. These results are in line with other publications on APRV in patients with ARDS.^7,8^ We postulate that the inverse ratio ventilation and increased inspiratory pressures open and stabilize derecruited alveoli especially in the dorsal lung regions thereby improving gas exchange and distribution of ventilation and perfusion through the pulmonary system. The positive effects on oxygenation were more pronounced for higher I: E and inspiratory pressures, which is consistent with the hypothesis that an open-lung strategy leads to improved gas exchange.

As expected, during APRV there was an increase in tidal volume. APRV is not a volume-controlled mode of ventilation and the increased pressure gradients, improved lung compliance secondary to recruitment and spontaneous breathing in non-paralyzed patients were all likely contributors to the increased tidal volumes. Interestingly, although the tidal volumes were increased, there was a decrease in total minute ventilation. The inspiratory and expiratory times during APRV were not available for our analysis. However, we can assume that the number of pressure releases per minute during APRV was less than the respiratory rate used during conventional mechanical ventilation and therefore the total minute ventilation during APRV was decreased.

Hypoventilation and hypercapnia are well known consequences of APRV. However, in our cohort there was a substantial and statistically significant decrease in PCO_2_ during APRV despite a decrease in total minute ventilation. Unfortunately, the ETCO_2_ before and during APRV was recorded for only 17 patients in our medical record system. Due to this limitation, we utilized the ventilatory ratio as an alternative surrogate of dead space which has been validated in previous studies.^4^ Our results demonstrated that APRV resulted in significant reduction in the ventilatory ratio indicating a reduction in dead space ventilation. We postulate that although the total minute ventilation was decreased, the effective alveolar ventilation and carbon dioxide clearance was improved during APRV as a result of improved alveolar recruitment and decreased dead space.

We studied a cohort of particularly compromised patients who presented with low P/F despite elevated PEEP and FIO_2_ and utilization of other rescue strategies including NMBAs, prone positioning and pulmonary vasodilators. Compared to survivors, patients who died were significantly older and presented with more profound hypoxemia and lower ventilatory ratio but there was no difference in the use of vasopressors, antibiotics or steroids. Interestingly, there was no difference in the plasma level of inflammatory markers, but non-survivors had a significantly higher WBC. This may reflect a superimposed bacterial infection as cause of further complications and death. In our cohort, during APRV trial, non-survivors had significantly higher airways pressure and a non-significant decrease in dynamic compliance (*P* = 0.055) compared to survivors, which may indicate severe lung damage with fibrotic changes and poor recruitability in this population. We noticed that survivors were intubated substantially earlier during their hospital course. Although the difference did not reach statistical significance (*P* = 0.0603) other authors have reported that early intubation may be associated with improved outcomes in patients with COVID-19.^9^

Our patients were transitioned to APRV late in their hospital course, and although we did not observe any difference in the time of APRV implementation between survivors and non survivors, it remains unclear if APRV can improve clinical outcomes when used as a primary mode of ventilation. To date, there are no prospective studies that have shown a mortality benefit in patients with ARDS treated with APRV. Zhou et al demonstrated that this mode of ventilation is associated with a significant decrease in length of stay and duration of mechanical ventilation when compared to the traditional low tidal volume ventilation strategy. Although there was a reduction in mortality, it did not reach statistical significance.^10^ Their study was limited by the low number of patients and was underpowered to determine the primary outcome. Given the multitude of proven physiological and clinical benefits, it is reasonable to hypothesize that an open lung strategy can potentially influence mortality in patients with ARDS and COVID-19. This hypothesis is further supported by a recent meta-analysis of patients with ARDS ventilated with APRV, which showed a decreased mortality with APRV compared to a low tidal volume strategy.^11^

APRV is a mode of ventilation that offers several advantages over conventional mechanical ventilation including preservation of spontaneous unassisted breathing and increased lung inflation time with improved alveolar recruitment and oxygenation.^2^

Despite these potential advantages, APRV remains an underutilized mode of ventilation in the intensive care units in North America.^12^ Possible reasons include, knowledge deficits related to the initiation and management of APRV, paucity of evidence from randomized control trials showing definitive clinical benefit and institutional policies.

In our institution at the Mount Sinai Health System, APRV is rarely used as a primary mode of ventilation. Instead, this mode of ventilation is mainly utilized as a last resort for refractory hypoxemia when other more conventional or evidence-based treatments such as prone positioning, pulmonary vasodilators, NMBAs and recruitment maneuver failed to achieve ventilation and oxygenation goals. As a result, our patients were transitioned to APRV late in their clinical course after conventional rescue therapies were attempted and respiratory status was already significantly compromised.

This study presents numerous limitations. First, it is a retrospective analysis from a single health system which limits the generalizability. However, it should be noted that the Mount Sinai Health System is a large integrated healthcare system encompassing 8 hospital campuses in the New York metropolitan area and serves a diverse patient population. Second, patient data was collected from a hospital database that relies on the accuracy of the health care personnel to document information in the electronic medical record. This becomes increasingly more challenging during a time of significant resource constraint such as that observed during the COVID-19 pandemic. Third, the small number of patients and missing data significantly affected the power of the study. This was particularly evident in the analysis of physiological variables between survivors and non survivors. In particular for our study, this included missing data on inspiratory and expiratory times during APRV, plateau pressures to calculate static lung compliance, and ETCO_2_ for the majority of patients. Finally, APRV initiation and settings were at the discretion of the practicing physician and varied considerably among the patients in our cohort.

## Conclusions

In patients with COVID-19 and refractory hypoxemia, APRV may improve alveolar recruitment, decrease dead space ventilation and equilibrate the distribution of ventilation and perfusion in different regions of the lungs resulting in improved oxygenation, alveolar ventilation and carbon dioxide clearance. These effects were more pronounced with higher airway pressure. This study contributes to the growing evidence on the positive effects of APRV on oxygenation and ventilation. Prospective studies are urgently needed to evaluate the potential benefits of APRV on clinical outcomes in patients with COVID-19 and severe hypoxemia.
